# A combined approach for genome wide protein function annotation/prediction

**DOI:** 10.1186/1477-5956-11-S1-S1

**Published:** 2013-11-07

**Authors:** Alfredo Benso, Stefano Di Carlo, Hafeez ur Rehman, Gianfranco Politano, Alessandro Savino, Prashanth Suravajhala

**Affiliations:** 1 Department of Control and Computer Engineering, Politecnico di Torino, I-10129 Torino, Italy; 2 Consorzio Interuniversitario Nazionale per l'Informatica, Verres (AO), Italy; 3 Bioclues Organization, ICICI Knowledge Park, Picket, Secunderabad 500011 AP, India

## Abstract

**Background:**

Today large scale genome sequencing technologies are uncovering an increasing amount of new genes and proteins, which remain uncharacterized. Experimental procedures for protein function prediction are low throughput by nature and thus can't be used to keep up with the rate at which new proteins are discovered. On the other hand, proteins are the prominent stakeholders in almost all biological processes, and therefore the need to precisely know their functions for a better understanding of the underlying biological mechanism is inevitable. The challenge of annotating uncharacterized proteins in functional genomics and biology in general motivates the use of computational techniques well orchestrated to accurately predict their functions.

**Methods:**

We propose a computational flow for the functional annotation of a protein able to assign the most probable functions to a protein by aggregating heterogeneous information. Considered information include: protein motifs, protein sequence similarity, and protein homology data gathered from interacting proteins, combined with data from highly similar non-interacting proteins (hereinafter called Similactors). Moreover, to increase the predictive power of our model we also compute and integrate term specific relationships among functional terms based on Gene Ontology (GO).

**Results:**

We tested our method on *Saccharomyces Cerevisiae *and *Homo sapiens *species proteins. The aggregation of different structural and functional evidence with GO relationships outperforms, in terms of precision and accuracy of prediction than the other methods reported in literature. The predicted precision and accuracy is 100% for more than half of the input set for both species; overall, we obtained 85.38% precision and 81.95% accuracy for *Homo sapiens *and 79.73% precision and 80.06% accuracy for *Saccharomyces Cerevisiae *species proteins.

## Background

Proteins are macromolecules that serve as building blocks and functional components of a cell, and account for the second largest fraction of the cellular weight after water. Proteins are responsible for some of the most important functions in an organism and the knowledge of their functions is a crucial link in the development of new drugs, better crops, and even the development of synthetic biochemicals such as biofuels. However, rapid advances in genome sequencing technologies are revealing new proteins at a rate that have resulted in a continually expanding sequence-function gap for the discovered proteins [1]. For example, in *Homo sapiens *more than half of the total proteins are uncharacterized, likewise about one-third of the proteins in the *Saccharomyces Cerevisiae*, which is arguably one of the most well characterized model organisms, remain functionally unknown.

This large set of conserved proteins whose function is still unknown, represents one of the main challenges for a deep comprehension of an organism as a biological system. Moreover, better understanding of protein functions can help biologists to successfully investigate new lines of attack against different diseases. Due to their enzymatic nature, proteins are generally among the preferred targets in drug and vaccine manufacturing processes. This makes the knowledge of their functions a critical step in any drug target discovery effort, and fully justifies the necessity of effective computational techniques for the precise annotation of uncharacterized proteins.

Until recently, numerous high-throughput experimental procedures have been developed to investigate the mechanisms leading to the accomplishment of a protein's function. Different information sources including sequence similarity, protein 3D structure, phylogenetic profiles, protein-protein interactions (PPI), gene expression profiles, protein complexes, etc., represent the ground for the development of these techniques [2]. The most widespread approaches utilize proteome-scale PPI networks that have been retrieved for several organisms including yeast and human [3], [4], [5], [6]. Interactions among proteins are mapped into graphs where each node signifies a protein and the edges between nodes represent associated molecular interactions of proteins. An interaction in the network is either a direct physical association between the proteins (typically retrieved via two hybrid analysis [7]), or a functional association in which the two interacting proteins are part of the same multi-protein complex, and cooperate for the same functional goal [8].

Protein function prediction methods that utilize protein interaction networks information can be categorized into three main groups: 1- *Module-assisted*, 2- *Direct methods*, and 3- *Probabilistic methods *[9]. Nevertheless, all methods share the common approach that tries to propagate protein annotations from functionally known proteins of a network to uncharacterized proteins [4].

*Module-assisted *methods search for protein modules of a network that are involved in a particular biological activity (i.e., versatile protein domains that are frequently used as building blocks in the construction of diverse multidomain proteins). Protein functional annotations are then assigned based on the presence of a protein in a specific module. Instead, *direct methods *are based on the fact that close proteins in the network are involved in related functional activity. Both direct neighbours [5] and indirect neighbours [10], try to establish functional links in the network by considering first or higher level interacting neighbor proteins.

Module-assisted and direct methods assume that proteins with similar functions are always close to each other in the network. However, this assumption can't be applied to every protein in the network [11]. To model such nature of proteins in the network, methods utilizing probabilistic frameworks based on Markov Random Fields (MRFs) are presented [12], [13], [14]. The fundamental supposition for such methods is that a protein's function is independent of all other proteins in the network given its neighboring proteins [9]. The techniques of this category, in general, estimate prior and conditional probabilities of all functions in the network and then approximate the joint probability of an unannotated protein to these functions.

The elusive nature of protein functions necessitates the use of appropriate function taxonomies to properly identify the set of activities a protein performs. Approaches that utilize PPI data coupled with a standard taxonomy of functions have demonstrated better results compared to those exploiting direct annotation transfers, as shown in [15], [16], [17], [18], and [19]. Most of these techniques use the Gene Ontology (GO) [20] as a functional classification scheme. GO is a structured and controlled vocabulary of terms providing consistency in annotating how a protein behaves in a cellular context. It is arranged in Directed Acyclic Graph (DAG) of nodes, associated in parent child relationships; with each node indicating a functional term. Nodes are connected with "is_a" (special case of the parent node/term) or "part_of" (sub-process of the parent node/term) relationships. Functionally known proteins are related to one or more nodes of the GO hierarchy; and because of parent/child associations if a protein is known to a child term it is also known to all of its parent terms in the hierarchy.

Several techniques have been proposed to use GO term relationships to functionally characterize proteins, e.g., [16], [21], [22] and [23]. Mitrofanova et al. [17] propose a Markov Random Field (MRF) based approach that integrates PPI networks with protein inter-species homology information considering a fixed size ontology. Unfortunately, while the fixed size ontology strongly reduces the computational complexity of the prediction process, it also represents one of the main limitations of this technique. This simplification limits the application of the methodology only to proteins annotated with the same fixed and specific set of GO terms. In fact, a method able to consider all functions of a protein along with their corresponding annotations in the whole GO would provide a more precise picture of the protein's cellular activity enabling for higher predictive power especially in the case of very large data sets of proteins.

Combining functional information from heterogeneous biological sources has also been proven to increase the overall predictive power of automated protein function annotation techniques [15], [19]. For a large set of uncharacterized proteins it is difficult to find enough biological information in PPI network databases for their functional association with other proteins. Moreover, existing interaction information is often unreliable, including a high rate of false positives. Heterogeneous information sources may provide additional functional links between uncharacterized proteins and annotated proteins.

**Figure 1:**
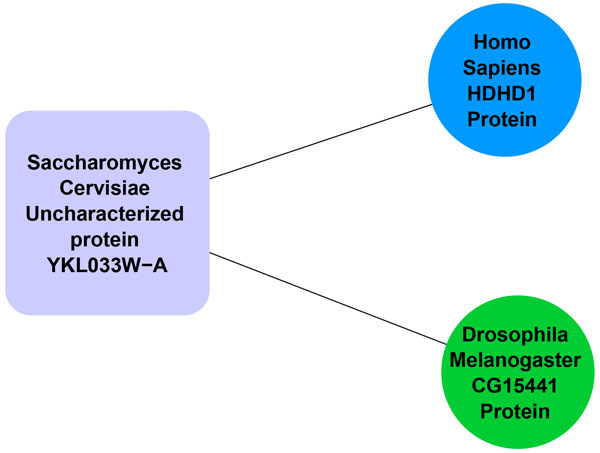
**An example of *Saccharomyces Cerevisiae *hypothetical protein connected with homolog proteins of other species networks**.

Protein homology among different species could be exploited for this purpose. Many hypothetical proteins show no interactions (i.e., no edges) in their own network, but are associated with high confidence edges to homologs of other species networks. An example of this type of association is shown in Figure 1. The protein *YKL033W-A *(UniProtID: Q86ZR7) of *Saccharomyces cerevisiae *does not show any interaction in its own network. Nevertheless, it has two interactions with high homolog similarity with protein *HDHD1 *(UniProtID: Q08623) of *Homo Sapiens *species and with protein *CG15441 *(UniProtID: Q94529) of *Drosophila Melanogaster *species networks. Another type of biological information that could be exploited to link characterized and uncharacterized proteins is the set of motifs conserved in those proteins. Several functionally conserved proteins are found to have motifs that associate them to a particular molecular activity. For example in Table 1, uncharacterized protein *YIL169C *(UniProtID: P40442) is conserved with Chemotaxis_Transduce_2 and T_SNARE motifs, while uncharacterized protein *Truncated TBY *(UniprotID: E9PAE3) is conserved with INTEGRASE and ASP_PROTEASE motifs. Similar motifs in known proteins can be used to link functional information with these proteins.

**Table 1:** An example of Baker Yeast's Hypothetical Proteins conserved with different motifs.

	Hypothetical Proteins	Motif Pattern and Profiles Conserved
1	YIL169C	Chemotaxis Transduce 2
		T SNARE
2	Truncated TYB	INTEGRASE
		ASP PROTEASE

This work is an extension of our previous work [18]; with the additional concept of network enrichment through similactor proteins which is particularly effective for proteins with relatively small network information. We present a novel high-throughput computational scheme for protein function prediction that aggregates heterogeneous biological information that can be retrieved for a large set of uncharacterized proteins. We build a computational model that integrates protein interaction data with sequence similarity, protein homolog similarity and protein shared motifs to calculate an interaction score exploited to measure the positive evidence of protein interactions and shared functions. The integrated model is then enriched with GO structural information to calculate a context similarity measure among potential protein annotations. The whole GO hierarchy is used without imposing restrictions on the set of considered GO terms, thus overcoming some of the limitations of [17]. The method yields high precision and accuracy over the previously reported methods with a wide protein coverage when applied to *Saccharomyces Cerevisiae *and *Homo sapiens *species proteins.

## Methods

**Figure 2:**
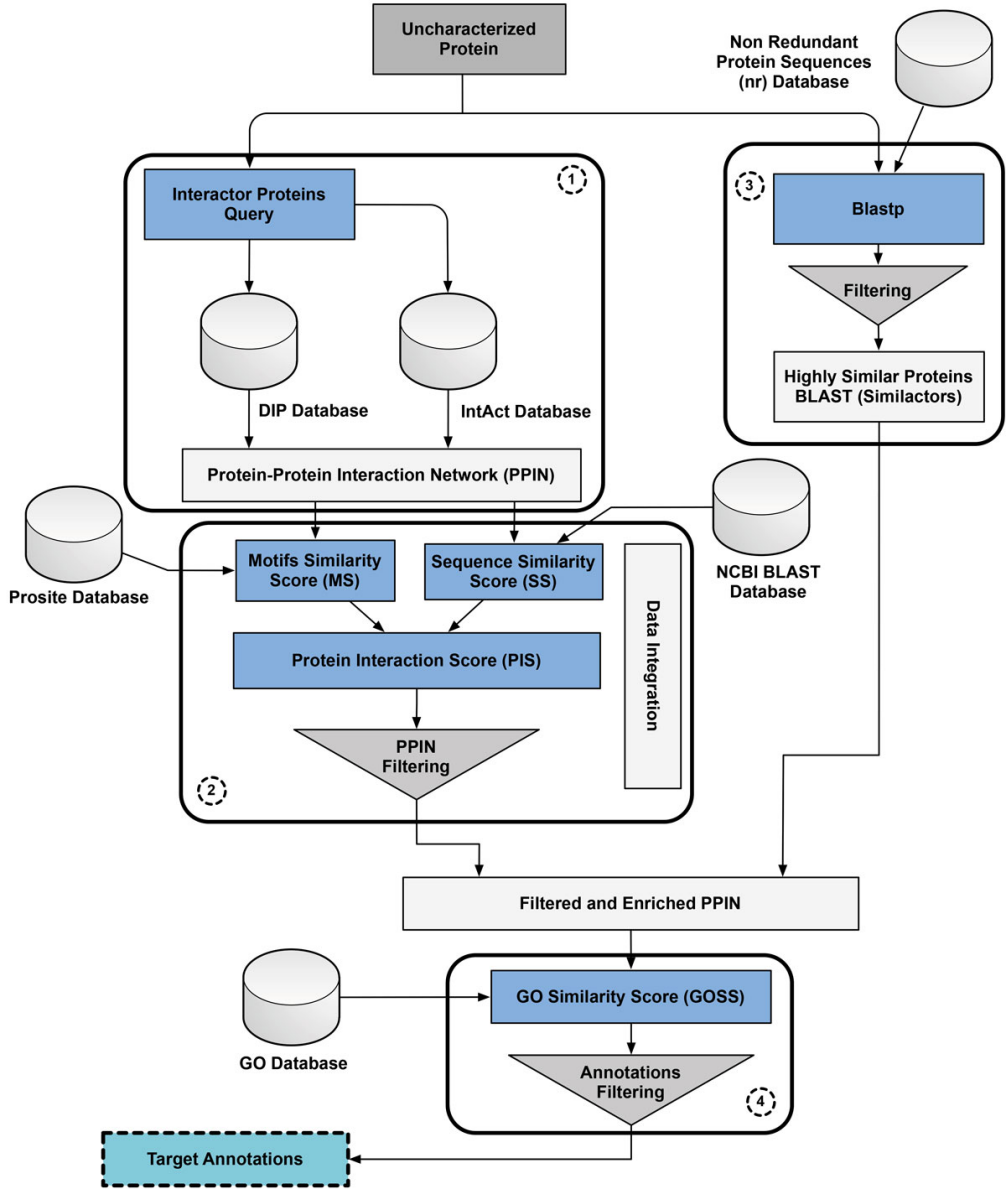
**High-level view of the information flow of the proposed protein annotation pipeline**.

Our protein annotation pipeline exploits the associative nature of proteins that interact and collaborate on a common biological activity. The functions of an uncharacterized protein can therefore be inferred when the functions of its binding or interacting partners is known. Figure 2 provides a general high-level view of the proposed information flow that comprises four main computational steps:

1. building the protein-protein interaction network,

2. filtering the network for reliable interactions,

3. enriching the network with a set of non interacting highly similar proteins, and

4. computing a GO based function similarity score to propagate functions from characterized to uncharacterized proteins.

### Building the PPI network

Given a target uncharacterized protein (*uP*) identified using its UniProt [24] identifier, the associated PPI data are queried from two well established protein interaction databases: (i) *IntAct*, a freely available, open source database system for molecular interaction data with all interactions derived from literature curation or direct user submissions [25], and (ii) *DIP *(Database of Interacting Proteins) [26], a database that documents experimentally determined protein-protein interactions and interaction networks in biological processes.

All non-redundant interacting proteins (*iP*) obtained from the two databases are used to build a protein- protein interaction network (PPIN) connecting *uP *with all identified interactors. Interaction data are acquired under different conditions and for different organisms. In particular, the study of the evolutionary relationships between species suggests that orthologs proteins that manifest high sequence similarity and whose functions have been established before speciation, are likely to share similar protein annotations. To capture homolog similarity based upon orthologs, the considered PPIN includes two sets of interacting proteins: (i) proteins of the same species of *uP*, and (ii) orthologs interacting proteins from other species.

It is worth mentioning here that the proposed methodology is not tied to the specific protein interaction databases considered in this paper. Additional databases or tools able to extract PPI information (e.g., Proteinquest [27]) can be exploited to enlarge the initial PPIN.

### Filtering the PPIN

Due to the heterogenous nature of protein interactions and to the type of experiments exploited to detect the interactions, PPI data are prone to false positives. Therefore, to increase the predictive power of the considered protein annotation flow, the initial PPIN must be properly filtered in order to keep reliable interactions, only. We therefore introduce a Protein Interaction Score (*PIS*) between two interacting proteins *uP *and *iP_j _*defined as follows:

(1) $$PIS\left(uP,iP_{j}\right)=MS\left(uP,iP_{j}\right)+SS\left(uP,iP_{j}\right),\forall iP_{j}\in PPIN$$

where *MS*(*uP, iP_j _*) ∈ [0, 1] measures the motif similarity of the two proteins, whereas *SS*(*uP, iP_j _*) ∈ [0, 1] measures the sequence similarity of the two proteins. Integrating different information in a single score is particularly important as each type of data typically captures distinct aspects of cellular activity.

Proteins often have several motifs with distinct evolutionary histories. The identification in the sequence of an annotated protein of patterns including evolutionarily conserved motifs can be associated to a specific biochemical function. Similar conserved motifs can be identified in the sequence of uncharacterized proteins, as well. Therefore, counting the number of common motifs conserved in two connected proteins represents a good opportunity to identify strong functional associations for uncharacterized proteins. Motif information has been taken into account gathering data from the ProSite database [28] and using them to introduce a motif similarity measure into our *PIS*. ProSite enables to query for a protein and to obtain a list of conserved motifs associated with a particular protein functional activity. We define the motif similarity score between *uP *and *iPj *as the number of common motifs conserved between the two interacting proteins (dividend of eq. 2) normalized to the minimum number of motifs obtained for the two proteins in isolation (divisor of eq. 2):

(2) $$MS\left(uP,iP_{j}\right)=\frac{\left|motif\left(uP\right)\cap motif\left(iP_{j}\right)\right|}{min\;\left(\left|motif\left(uP\right)|,|motif\left(iP_{j}\right)\right|\right)},\forall iP_{j}\in PPIN$$

High scores indicate proteins sharing several conserved motifs and therefore with higher possibility of sharing the same function. Experimental results showed that *MS *is in general biased toward either 0 (i.e., no motifs are shared) or 1 (all motif are shared).

The second measure that contributes to increase the PIS is the sequence similarity. Sequence similarity between two proteins is a strong hint for interaction relevance. Proteins with highly similar sequences are found to have been involved in similar functional activities. To capture sequence similarity between proteins we therefore define a sequence similarity score between protein *uP *and protein *iP_j _*as a normalized pairwise BLAST score [29]. The BLAST algorithm is a sequence comparison algorithm that is optimized for speed and used to search sequence databases for optimal local alignments to a query and a BLAST score of two proteins is a number that denotes the overall significance of a sequence alignment between two protein sequences. High scores correspond to high similarity. We use a normalized BLAST score, defined as the BLAST score of the two proteins divided by the self score of the query (i.e., the BLAST score of the protein against itself), as reported in eq. 3.

(3) $$SS\left(uP,iP_{j}\right)=\frac{BLAST\left(uP,iP_{j}\right)}{BLAST\left(uP\right)},\forall iP_{j}\in PPIN$$

Interacting nodes with high *PIS *are more likely to correspond to reliable interactions and therefore to identify proteins that actually participate in common functions. A threshold *PIS_th _*is used to filter low scored interactions from the PPIN, and to identify a reliable set of interacting proteins. Different threshold values have been investigated in the performed experiments (i.e., *PIS_min _*∈ {0,0.25,0.5}). Following [17], if no shared motifs are identified (i.e., *MS *= 0), proteins with less than 50% sequence similarity (*SS <*0.5) are good candidates to be discarded motivating the maximum considered threshold of 0.5. If shared motifs are identified, also proteins with less than 50% sequence similarity can be still considered as valid interactors.

### PPIN enrichment through similactor proteins

Together with a large set of false positives, PPI information are also prone to false negatives (i.e., unknown or missing interactions). This is due to the fact that a large fraction of uncharacterized proteins are only known with their amino acid sequences. Sequence information can be used to enrich the filtered PPIN with additional interactions with other known proteins through sequence alignment.

We use the *blastp *[30] tool of NCBI (National Center for Biotechnology Information), which is designed to find local regions of similarity with target database sequences, to BLAST *uP *against all sequences contained in the *Non Redundant Protein Sequences *(*nr *) database [29] and to obtain a list of highly aligned protein sequences. The *nr *database compiled by the NCBI is one of the largest and most prominent databases that accumulates and stores almost all the available protein sequences. It contains non-redundant sequences from GenBank, CDS translations, PDB, Swiss-Prot, PIR, and PRF. If the similarity of *uP *spans the whole sequence, *blastp *also accounts a global alignment, which is the preferred score used to rank the sequence similarity.

Enriching the PPIN with additional proteins may, on the one hand, reduce the number of false negatives, but, on the other hand, it can introduce new false positive interactions. It must be therefore limited to a very small set of proteins that show very high similarity. In the experiments performed in this paper, for each *uP *, only the first 10 ranked non-interacting (i.e., not already identified in the PPIN building phase) highly similar proteins (Similactors) out of the full set of proteins returned after alignment from the *nr *database has been considered for the PPIN enrichment. This set has been further filtered removing all PDB structures and uncharacterized proteins in order to enrich the PPIN with a very small set of reliable similactors.

### Similarity scores based on gene ontology

The set of interacting proteins available in the filtered and enriched PPIN defines the set of candidate functions for *uP *. Functions are represented according to the GO taxonomy as GO terms (i.e., nodes of the ontology). However, GO is organized into three principle ontologies namely: molecular function, biological process and cellular component, whereas each ontology is structured in a DAG of terms. Each term is therefore part of a GO hierarchy. For our scheme we focus on the molecular function GO hierarchy, which describes activities performed by a protein at the molecular level. This is particularly important to understand the gene product in detail. Since GO nodes are connected to other nodes through parent-child relationships, and a protein known to a term in GO is also known to all the parent terms of the hierarchy, we can represent each annotation of an interacting protein *iP *(denoted as $A_{i}^{iP_{j}}$ with its full GO molecular function hierarchy. $A_{i}^{iP_{j}}$ is therefore an ordered list of GO terms starting from the specific node identifying a specific function and including all nodes to traverse before reaching the top of the hierarchy (the root is not included in this set).

Given this definition of annotation, it is possible to compute a GO similarity score (GOSS) between two annotations of two different proteins on the basis of their relative positioning in the GO hierarchy according to eq. 4. The dividend of eq. 4 measures how much the two annotations overlap, counting the number of common terms in the hierarchy. The divisor of eq. 4 normalizes the overlapping to the hierarchy size of the shortest annotation.

(4) $$GOSS\left(A_{i}^{iP_{j}},A_{z}^{iP_{k}}\right)=\frac{\left|A_{i}^{iP_{j}}\cap A_{z}^{iP_{k}}\right|}{\text{min}\left(\left|A_{i}^{iP_{j}}|,|A_{z}^{iP_{k}}\right|\right)}$$

GOSS is computed for all couples of annotations and proteins available in the filtered and enriched PPIN resorting to GO structural data downloaded from the GO database [20] for the molecular function class hierarchy. To reduce the computational effort, only couples of annotations in which the top term is equal (i.e., they belong to the same functional context) are considered. For all other terms, since the two annotations belong to different contexts, the GOSS can be directly set to zero. Once all scores have been computed, a threshold (*GOSS_th_*) is used to filter GOSS results and to select those annotations that likely represent a valid function for *uP*. For all scores that cross the threshold, the minimum length hierarchy annotation out of the two that have been compared during the score calculation is selected and used to annotate *uP*. The shortest annotation is selected because up to that level the molecular activity of interacting proteins is certain.

### Protein annotation example

**Figure 3:**
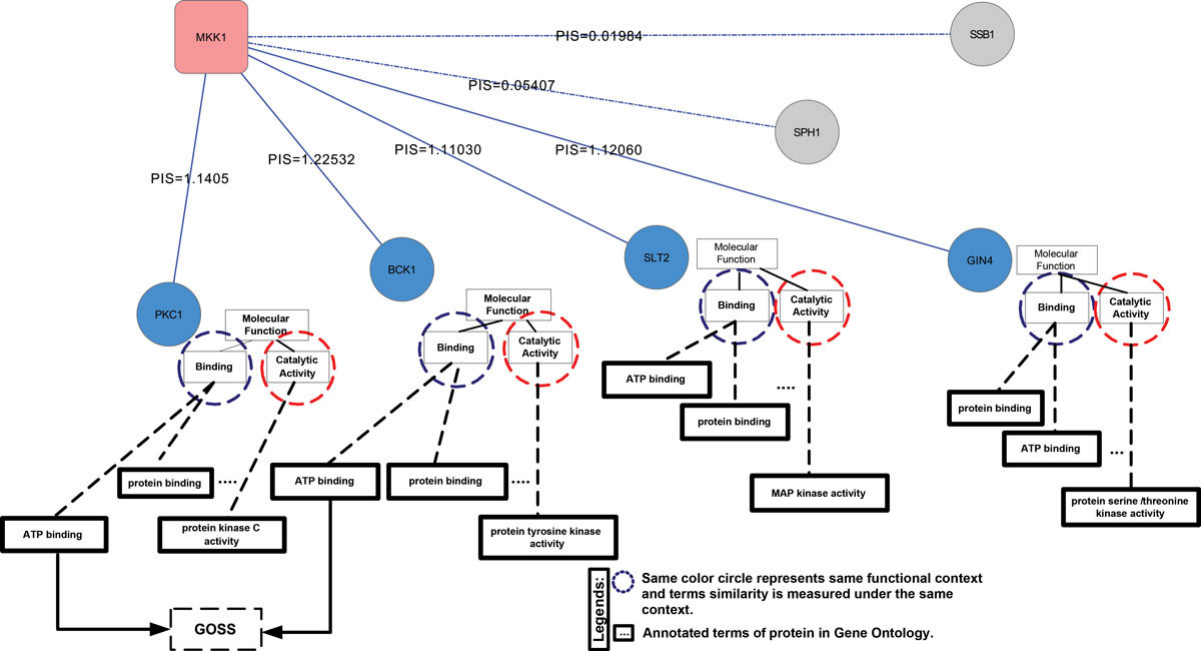
**An example of context similarity score based on Gene Ontology for *MAP kinase kinase MKK1/SSP32 *protein**.

To help understanding the computational steps involved in the annotation of a protein we consider the example of *MAP kinase kinase MKK1/SSP32 *protein (*MKK1*, UniProtID P32490), which is annotated in the UniProt database with three molecular functions: *(1) ATP binding, (2) Protein binding*, and *(3) Protein serine/threonine Kinase activity *functions. We assume *MKK1 *to be our target uncharacterized protein and we try to predict its functions using our scheme, as reported in Figure 3.

*MKK1 *has 21 interactors obtained from IntAct and DIP databases resulting in a PPIN network of 22 nodes with a subset of them depicted in Figure 3. For each edge of the PPIN, we calculated the PIS according to eq. 1 and we filtered the interactions applying a threshold of *PISth *= 0.5, obtaining a set of 4 candidate interactors: (1) *Protein Kinase C-Like1 *(*PKC1*, UniProtID P24583), (2) *Serine/threonine-protein kinase BCK1/SLK1/SSP31 *(*BCK1*, UniProdID Q01389), (3) *Mitogen-activated protein kinase SLT2/MPK1 *(SLT2, UniProtID Q00772), and (4) *Serine/threonine-protein kinase GIN4 *(GIN4, UniProtID Q12263). For the sake of simplicity no similactors are included in this example.

Potential interactors are annotated with a number of functions. We map them on GO terms to obtain related term dependencies as shown in Figure 3. In our example, there are only two functional contexts among all interactors of MKK1 namely, *binding*, and *catalytic activity*. For protein annotations under the same functional context, we computed the GOSS score according to eq. 4. For instance, let us compare the *ATP binding *annotation of protein *PKC1 *$\left(A_{ATP-binding}^{PKC1}\right)$ and the *ATP binding *annotation of protein *BCK1 *$\left(A_{ATP-binding}^{PKC1}\right),$ under the *binding *context. Since both proteins are annotated with the same GO terms (i.e., all terms in the hierarchy overlap) and this term is elaborated at the sixth level in the GO hierarchy, according to eq. 4 *GOSS *$(A_{ATP-binding}^{PKC1},\;A_{ATP-binding}^{PKC1})=6/6=1.$ GOSS equal to 1 means one of the term is completely part of the other. For *MKK1 *both interacting proteins *PKC1 *and *BCK1 *are found to be involved in *ATP binding activity *with high GO similarity. We therefore annotate protein *MKK1 *with this functional term. Likewise, we calculate GOSS for other couples of annotations of the interacting proteins and obtain two additional valid annotations *Protein binding*, and *Protein serine/threonine Kinase activity *with high GO similarity compared to other terms. In summary, all original annotations of *MKK1 *have been properly predicted by the proposed protein annotation flow.

## Results and discussion

To validate the pipeline described in the Methods section, we applied the annotation process to predict the functions of two *Saccharomyces Cerevisiae *and *Homo sapiens *species protein datasets. The protein functional annotation data used for our model were obtained from the Uniprot [24] database for both species, and the functional term-related dependencies were extracted from the GO database [20]. To calculate the prediction performance we used a leave-one-out cross-validation approach: each annotated protein *P *in our dataset has been selected as a candidate unknown protein and its functional annotations predicted resorting to our methodology. Predicted functions have been then compared with the protein's original annotations in order to understand the overall prediction performance. The process has also been repeated under several different thresholds settings. We present the results for 763 proteins annotated with 2,099 GO terms of *Saccharomyces Cerevisiae *species, and 793 proteins annotated with 2,178 GO terms of *Homo sapiens *species.

### Performance evaluation metrics

Conceptually, protein activities are very much related to each other. To precisely understand and evaluate the proposed experimental results it is necessary to provide a clear definition of how True Positive (TP), True Negative (TN), False Positive (FP) and False Negative (FN) predictions are defined. These definitions may significantly impact not only the overall statistical strength of the experiments, but also the comparison with other methods.

**Figure 4:**
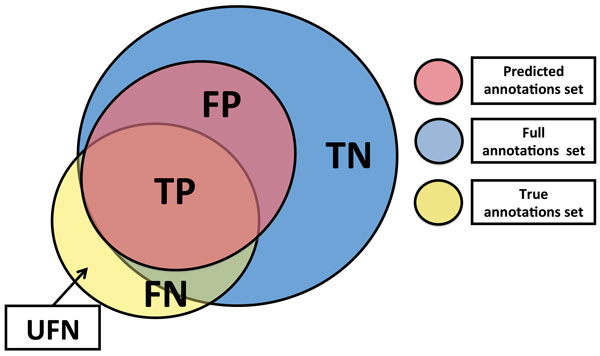
**Schematic view of predicted annotations, true annotations, and full annotation set, along with the concept of unpredictable FN**.

Figure 4 provides a graphical view of the different ways annotations for a target protein *P *under test can be classified. Three main sets of annotations can be defined:

1. *True annotation set*: the set of GO terms for which the target protein is actually annotated in UniProt. It represents the reference set to which our predictions can be compared;

2. *Full annotations set*: is the set of all GO terms found in the annotations of all interactors extracted for the target protein (before applying the filtering) plus the annotations of all selected Similactors. This represents the actual set of functions that our method is able to predict for a given protein.

3. *Predicted annotations set*: is the set of GO terms predicted by the proposed computational model for the selected protein.

Based on these three sets of annotations, our predictions can be classified as follows:

• TP terms are the intersection between the predicted annotations (i.e., predicted annotation set) and the real annotations (i.e., true annotation set). It is important to remember here that nodes in GO are hierarchically arranged from the most abstract term to the more detailed levels of activities and, if a protein is annotated to a child, it is also annotated to its parent. Therefore, annotating a protein with the functional activity of the parent node in the GO hierarchy may be considered as a TP. Nevertheless, methods that consider the prediction to parent terms as TP actually reduce the protein annotation information. Therefore, their prediction strength cannot be fairly compared with those methods that aim at predicting only the actual annotations of the proteins. Our results are compiled by considering as TP only those terms that correspond exactly to one of the true annotations. All other predictions are considered as false positives.

• FP terms are all terms in the predicted annotations set excluding the previously defined TP terms.

• FN terms are those functions that are present in the true annotations set but are not present in the predicted annotations set (i.e., missing predictions). Within the FN set, it is possible to identify a subset of annotations that we call Unpredictable False Negatives (UFN). It corresponds to the set of true functions of the test protein that are not part of the full annotation set. The UFN terms cannot be strictly called false negatives because our method does not actually reject those annotations. The bottleneck is rather to find enough biological information that could be used to include interacting proteins annotated with those functions in order to include them in the full annotations set. However, in order to provide worst case results, UFN have been included in the computation of FN.

• TN terms are all the terms in the full annotations space excluding the predicted true terms.

We computed TP, TN, FP and FN for each of the 1,556 proteins composing the two considered datasets. Cumulative TP, TN, FP and FN for each dataset have been then used to compute the following set of performance measures:

(5) $$\begin{array}{ccc} precision & = & \frac{TP}{TP+FP}\\ recall & = & \frac{TP}{TP+FN}\\ accuracy & = & \frac{TP+TN}{TP+TN+FP+FN}\\ F1 & = & \frac{2\cdot precision\cdot recall}{precision+recall} \end{array}$$

### Performance results

Table 2 reports the performance metrics for the two data-sets computed with different *PIS_th _*thresholds and with *GOSS_th _*= 0.99.

**Table 2:** Precision, Recall, Accuracy, and F1 for *S. Cerevisiae* and *Homo sapiens* datasets under different PISth and high GOSS values

*PISth*	*GOSSth*	Dataset	Precision %	Recall %	Accuracy %	F1 %
0.0	0.99	Homo sapiens	29.05	88.30	77.50	43.72
		S. Cerevisiae	05.78	92.92	66.16	10.87
0.25	0.99	Homo sapiens	83.34	79.71	82.65	81.48
		S. Cerevisiae	75.30	86.56	81.16	79.21
0.50	0.99	Homo sapiens	85.38	79.11	81.95	82.12
		S. Cerevisiae	79.73	81.76	80.06	80.73

Cerevisiae and Homo sapiens datasets under different *PISth *and high GOSS values

Results show that combining the filtering capability of the *PIS *with the introduction of similactors in the PPIN significantly improves precision, accuracy, and FP rate. This can be appreciated by comparing these results with the ones published for *Homo sapiens *species in [31].

To understand the effect *PIS_th _*on the overall prediction performance, experiments have been repeated with three different thresholds: *PIS_th _*= 0.5, *PIS_th _*= 0.25 and *PIS_th _*= 0. High values of *PIS_th _*guarantee better performances in terms of precision, accuracy, and F1, since the algorithm only selects highly reliable interactors. Reducing *PIS_th _*results in downgraded precision and accuracy values for both species. This is caused by the fact that a lower *PIS_th _*means selecting all interactors of the test protein along with its similactors as potential proteins. This leads to a too heterogeneous set of potential annotations and consequent lower precision and accuracy.

The presented results were compiled including UFN terms, i.e., considering UFN as FN. However, the inclusion of UFN terms in the analysis does not render the complete predictive strength of the experiments. Table 3 reports the results of the same experiments but including only predictable false negative terms for both species. Clearly, the accuracy and recall values show a significant increase.

**Table 3:** Precision, Recall, Accuracy, and F1 for *S. Cerevisiae* and *Homo sapiens* datasets without UFN terms

*PISth*	*GOSSth*	Dataset	Precision %	Recall %	Accuracy %	F1 %
0.0	0.99	Homo sapiens	29.05	94.375	78.05	44.42
		S. Cerevisiae	05.78	95.85	66.20	10.89
0.25	0.99	Homo sapiens	83.34	90.84	87.80	86.93
		S. Cerevisiae	75.30	90.81	84.04	82.33
0.50	0.99	Homo sapiens	85.38	90.35	87.66	87.79
		S. Cerevisiae	79.73	89.44	83.73	84.31

Cerevisiae and Homo sapiens datasets without UFN terms.

To better evaluate the contribution of the GO-based annotation transfer, we repeated the experiments using a direct annotation, i.e., *GOSS_th _*= 0. This means directly annotating the test protein with all functions of the identified potential interacting proteins. Results are shown in Table 4. Except for the recall, the GO based annotation transfer shows superiority in all metrics. The GO-based annotation transfer increases the number of TN terms, and strongly decreases the number of FP terms, which consequently leads to higher precision and accuracy.

**Table 4:** Comparison of results with and without GO based relationships

Dataset	*PISth*		Precision	Recall	Accuracy	F1
Homo sapiens	0.0	with GO	29.05	94.375	78.05	44.42
		w/o GO	11.49	99.39	13.79	20.60
S. Cerevisiae	0.0	with GO	05.78	95.85	66.20	10.89
		w/o GO	02.30	97.47	02.89	04.51
Homo sapiens	0.25	with GO	83.34	90.84	87.80	86.93
		w/o GO	60.24	97.65	63.51	74.51
S. Cerevisiae	0.25	with GO	75.30	90.81	84.04	82.33
		w/o GO	52.58	96.17	56.12	67.99
Homo sapiens	0.50	with GO	85.38	90.35	87.66	87.79
		w/o GO	65.22	97.22	67.38	78.06
S. Cerevisiae	0.50	with GO	79.73	89.44	83.73	84.31
		w/o GO	64.17	94.91	65.84	76.57

Comparison of results with and without GO based relationships

Another important observation is that the use of the GOSS similarity measure enables to decrease the False Positive Rate (FPR) for both data sets with increasing similarity values. We calculate the FPR as:

(6) $$FPR=\frac{FP}{TN+FP}$$

**Figure 5:**
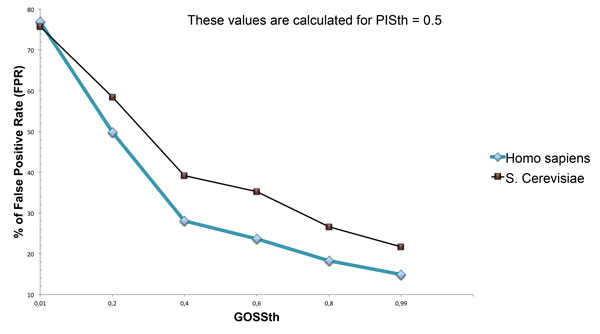
**False Positive Rate trend for both Homo sapiens and Saccharomyces Cerevisiae datasets**.

The FPR is decreased from 77% to 14% for Homo sapiens dataset and from 76% to 21% for Saccharomyces Cerevisiae dataset as shown in Figure 5. This result demonstrates that, thanks to GO-based similarity, predictions are more centered towards a semantically related annotation set.

### Comparison with other approaches

**Figure 6:**
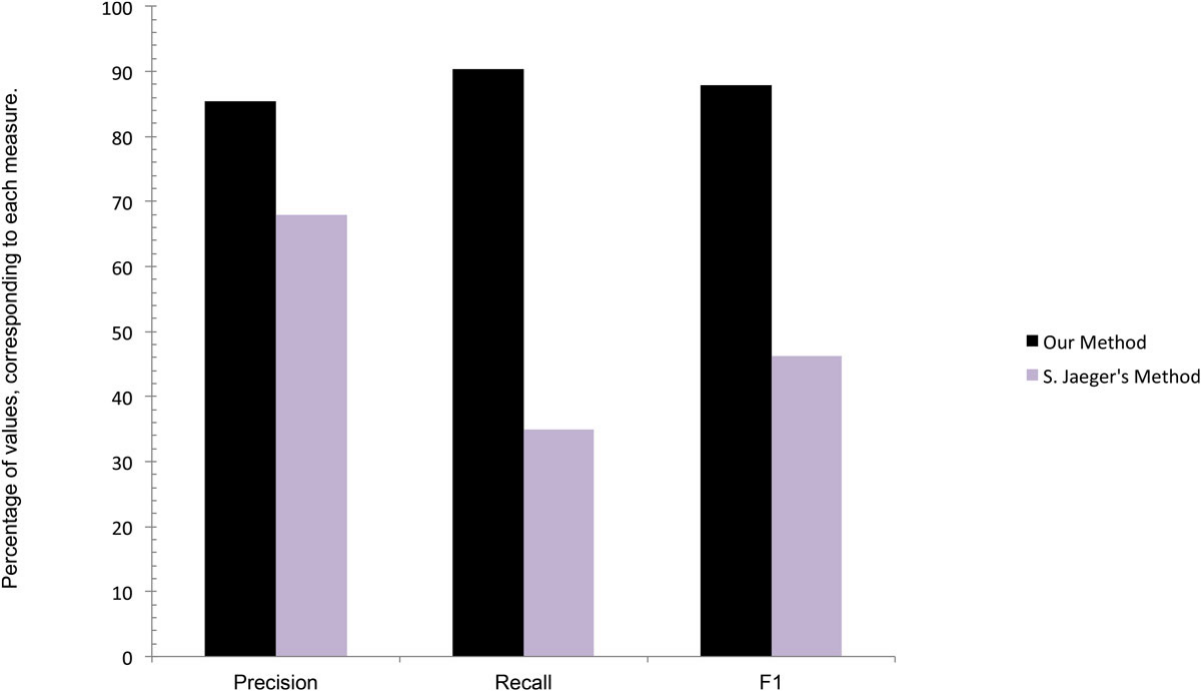
**Comparison of Precision, Recall, and F1 of our method (black) with S.Jaeger's [32], method (purple)**.

In this section, we compare our method with other techniques that also integrate multiple sources of information for protein annotation. The first technique we compare with is presented by S. Jaeger's et al. [32]. This technique proposes a scheme for predicting functional annotations of proteins by comparing interaction networks from various species and by utilizing orthology relationships, conserved modules and local PPI neighborhoods. It incorporates PPI data from various databases, and detects maximal conserved and connected sub-graphs in the interaction sets using approximate cross-species network comparisons. Finally, predictions are made for proteins within functionally coherent connected sub-graphs. The predictive strength of our technique can be compared with this technique, since it reports the function prediction results for our same *Homo sapiens *dataset. The results reported in Figure 6 show how our method outperforms the other in precision, recall, and F1 scores. This enhanced performance can be attributed to both the ability of our algorithm to identify functionally similar proteins, and to the use of the GO-based similarity measures to increase TP, TN terms and to reduce FP terms.

**Figure 7:**
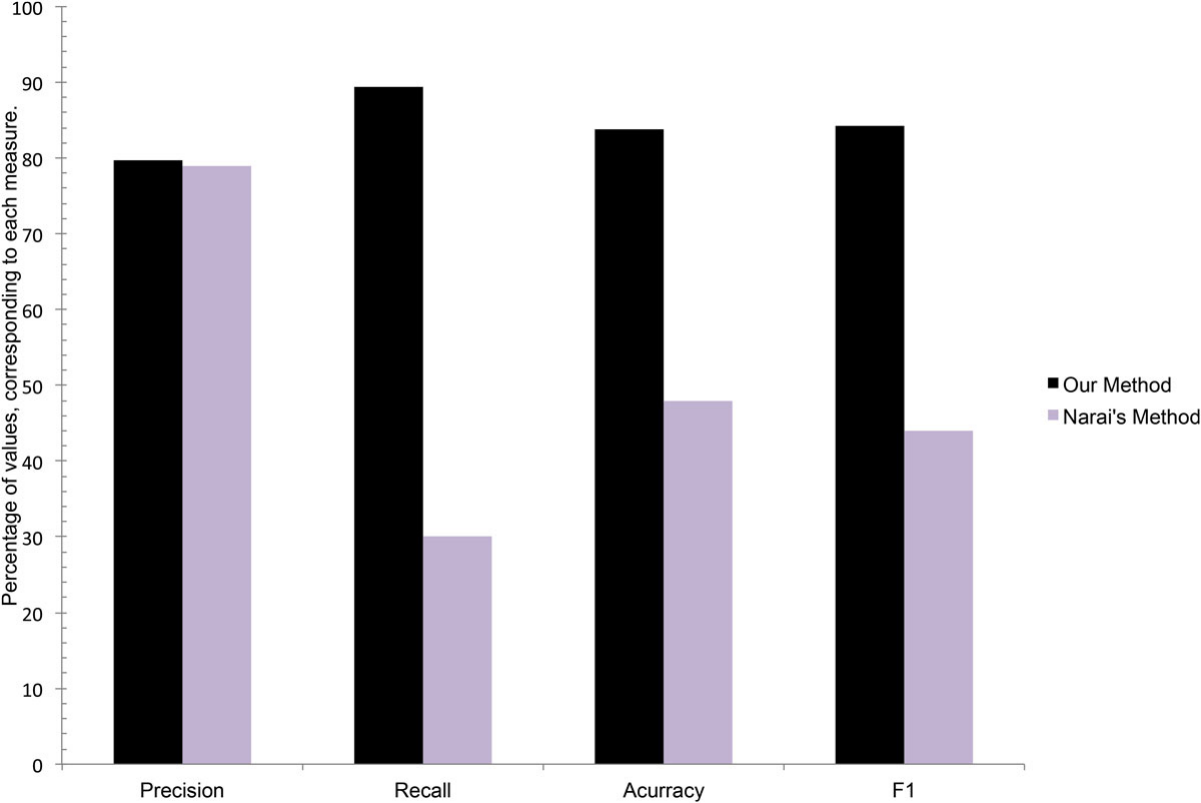
**Comparison of Precision, Recall, Accuracy and F1 of our method (black) with Narai's [15] method (purple)**.

Another successful technique has been proposed by Nariai et al. [15]. This method proposes and evaluates a probabilistic approach for protein function prediction that incorporates heterogeneous data. The association among proteins is established by means of interaction graphs constructed from PPI and gene expression data. The scheme is based on the assumption that neighboring proteins are more likely to share functions, compared to proteins that are not neighbors. The interaction graphs along with protein domain, mutant phenotype and protein localization data are integrated into a probabilistic Bayesian framework, which accordingly assigns a probability to each protein in the network representing the likelihood of positive or negative annotation to a specific function [15]. We compared Narai's best prediction results, i.e., the ones with optimum values of precision and accuracy, with our results for *Saccharomyces Cerevisiae *proteins providing prediction performance indicators for both methods in Figure 7. Regardless the considered performance indicator our method provides higher prediction capability compared to Narai's method. In particular we have been able to strongly reduce the false negative rate compared to the Narai's approach thus obtaining significant improvements in the prediction recall. A significant improvement in the true negative rate coupled with the reduction of the false negative rate also allowed us to outperform Narai's method in terms of prediction accuracy.

Finally, we compare our results with a more recent technique proposed by A. Mitrofanova et al. [17]. In this method the authors present a novel probabilistic chain-graph-based approach for predicting protein functions that builds on connecting networks of two different species by links of high interspecies sequence homology. The model is further enhanced to account for the GO based dependencies by linking multiple but related functional ontology categories within and across multiple species. Although the results reported by this approach show a very high Precision, Recall, Accuracy, and F1, from the perspective of the number of predicted functions, our technique is able to predict a larger set of individual GO terms with 100% precision. Nevertheless, a direct comparison of our measures with Mitrofanova's ones is not possible. Mitrofanova's approach operates on fixed size ontologies (8, 12, and 16 GO terms), whereas our method is independent of the ontology size. We have no way to elaborate on how their method would perform for larger ontology sizes and increased complexity. It is important to consider that limiting ontology size also limits the proteins annotated to it. Therefore, the protein dataset for cross validation is different in the two methods; in our case the set is larger and with higher diversity in annotations.

### Term wise prediction results

To complete the evaluation of our proposed methodology, we report a set of measures on the coverage of the GO terms that appear in the cross validation test of our protein datasets. The complete term wise prediction results can be seen in the supplemental material (Additional file 1 & Additional file 2).

For each functional term in the GO hierarchy we report

• *the Total Appearance Count*, which is the number of proteins in the dataset that are annotated with that functional term;

• the *Total Prediction Count*, which is the number of times that a term has been correctly predicted;

• the *Term Coverage*, which is the percentage of *Total Predictions *over the *Total Appearance *of each term.

For 330 unique GO terms appearing in the annotation of the dataset for *Homo sapiens *species we predicted 201 terms with 100% precision; likewise for 263 unique GO terms for *Saccharomyces Cerevisiae *species we predicted 165 terms with 100% precision.

## Conclusion

In this work, we presented a methodology that uses existing biological data with Gene Ontology functional dependencies to infer functions of uncharacterized proteins. We combined different sources of structural and functional information along with Gene Ontology relationships to predict multiple but related functional categories of unannotated proteins. These term-specific relationships, defined to clearly identify the functional contexts of activity of the interacting proteins, enables a dramatical improvement of the annotation accuracy with respect to previous approaches. The presented methodology may be easily extended to integrate more sources of biological information to further improve the function prediction confidence.

## Competing interests

The authors declare that they have no competing interests.

## Supplementary Material

Additional file 1 **Term wise prediction-Homo sapiens.pdf **Term wise prediction results for ***Homo sapiens ***data set. Click here for file

Additional file 2 **Term wise prediction-Cerevisiae.pdf **Term wise prediction results for ***Saccharomyces Cerevisiae ***data set. Click here for file

## Declarations

The research activity described in this paper been partially supported by Grant No. CUP B15G13000010006 awarded by the Regione Valle d'Aosta for the project: "Open Health Care Network Analysis". Politecnico di Torino directly supported all the related publication costs.

This article has been published as part of *Proteome Science *Volume 11 Supplement 1, 2013: Selected articles from the IEEE International Conference on Bioinformatics and Biomedicine 2012: Proteome Science. The full contents of the supplement are available online at http://www.proteomesci.com/supplements/11/S1.
