# Supplementary material for: A combined approach for genome wide protein function annotation/prediction
Source: Proteome Sci. 2013 Nov 7;11(Suppl 1):S1. doi: 10.1186/1477-5956-11-S1-S1 (PMC3909112; doi:10.1186/1477-5956-11-S1-S1)
Supplement: Additional file 1 — Term wise prediction-Homo sapiens.pdf Term wise prediction results for Homo sapiens data set. [file 1477-5956-11-S1-S1-S1.pdf]

| Gene Ontology Function | Total Appearance Count | Total Prediction Count | Term Coverage |
|---|---|---|---|
| **GO:0005008** | 1 | 1 | 100.0% |
| **GO:0005524** | 234 | 234 | 100.0% |
| **GO:0004713** | 18 | 11 | 61.1111111111% |
| **GO:0004715** | 10 | 7 | 70.0% |
| **GO:0003677** | 104 | 71 | 68.2692307692% |
| **GO:0003950** | 3 | 3 | 100.0% |
| **GO:0008270** | 127 | 125 | 98.4251968504% |
| **GO:0051287** | 6 | 6 | 100.0% |
| **GO:0004674** | 79 | 77 | 97.4683544304% |
| **GO:0005057** | 2 | 1 | 50.0% |
| **GO:0046872** | 34 | 34 | 100.0% |
| **GO:0002020** | 1 | 0 | 0.0% |
| **GO:0015267** | 2 | 2 | 100.0% |
| **GO:0005201** | 13 | 13 | 100.0% |
| **GO:0005509** | 25 | 24 | 96.0% |
| **GO:0005198** | 14 | 13 | 92.8571428571% |
| **GO:0043208** | 1 | 1 | 100.0% |
| **GO:0004707** | 6 | 5 | 83.3333333333% |
| **GO:0008353** | 5 | 3 | 60.0% |
| **GO:0004714** | 5 | 4 | 80.0% |
| **GO:0005003** | 1 | 1 | 100.0% |
| **GO:0004197** | 10 | 10 | 100.0% |
| **GO:0019899** | 1 | 0 | 0.0% |
| **GO:0030235** | 3 | 1 | 33.3333333333% |
| **GO:0000981** | 2 | 2 | 100.0% |
| **GO:0004675** | 2 | 0 | 0.0% |
| **GO:0005024** | 4 | 4 | 100.0% |
| **GO:0046332** | 3 | 0 | 0.0% |
| **GO:0004702** | 3 | 3 | 100.0% |
| **GO:0016361** | 2 | 0 | 0.0% |
| **GO:0005025** | 1 | 0 | 0.0% |
| **GO:0008022** | 2 | 0 | 0.0% |
| **GO:0004697** | 5 | 5 | 100.0% |
| **GO:0004190** | 2 | 1 | 50.0% |
| **GO:0004861** | 1 | 1 | 100.0% |
| **GO:0004716** | 2 | 2 | 100.0% |
| **GO:0004221** | 17 | 17 | 100.0% |
| **GO:0004843** | 6 | 6 | 100.0% |
| **GO:0000405** | 2 | 2 | 100.0% |
| **GO:0003697** | 8 | 8 | 100.0% |
| **GO:0004003** | 6 | 3 | 50.0% |
| **GO:0009378** | 2 | 2 | 100.0% |
| **GO:0043140** | 2 | 2 | 100.0% |
| **GO:0051880** | 2 | 2 | 100.0% |
| **GO:0000339** | 1 | 0 | 0.0% |
| **GO:0003729** | 6 | 3 | 50.0% |
| **GO:0003743** | 6 | 6 | 100.0% |
| **GO:0004386** | 7 | 6 | 85.7142857143% |
| **GO:0008026** | 10 | 10 | 100.0% |
| **GO:0008135** | 3 | 0 | 0.0% |
| **GO:0051082** | 3 | 2 | 66.6666666667% |
| **GO:0003700** | 66 | 50 | 75.7575757576% |
| **GO:0003779** | 2 | 2 | 100.0% |
| **GO:0005200** | 16 | 12 | 75.0% |

| | | | |
|---|---|---|---|
| **GO:0005543** | 10 | 10 | 100.0% |
| **GO:0030506** | 1 | 0 | 0.0% |
| **GO:0004699** | 1 | 1 | 100.0% |
| **GO:0004871** | 13 | 10 | 76.9230769231% |
| **GO:0008047** | 1 | 1 | 100.0% |
| **GO:0030546** | 1 | 1 | 100.0% |
| **GO:0035276** | 1 | 1 | 100.0% |
| **GO:0005011** | 1 | 0 | 0.0% |
| **GO:0043560** | 1 | 0 | 0.0% |
| **GO:0004867** | 1 | 1 | 100.0% |
| **GO:0008201** | 3 | 3 | 100.0% |
| **GO:0046914** | 2 | 2 | 100.0% |
| **GO:0008289** | 4 | 3 | 75.0% |
| **GO:0042803** | 10 | 5 | 50.0% |
| **GO:0051434** | 1 | 1 | 100.0% |
| **GO:0003725** | 4 | 4 | 100.0% |
| **GO:0004004** | 3 | 0 | 0.0% |
| **GO:0001047** | 1 | 0 | 0.0% |
| **GO:0003682** | 39 | 30 | 76.9230769231% |
| **GO:0003713** | 8 | 8 | 100.0% |
| **GO:0003823** | 12 | 10 | 83.3333333333% |
| **GO:0004402** | 2 | 2 | 100.0% |
| **GO:0004468** | 2 | 1 | 50.0% |
| **GO:0004842** | 19 | 19 | 100.0% |
| **GO:0008545** | 2 | 1 | 50.0% |
| **GO:0003714** | 3 | 2 | 66.6666666667% |
| **GO:0004672** | 15 | 7 | 46.6666666667% |
| **GO:0043565** | 42 | 39 | 92.8571428571% |
| **GO:0016874** | 6 | 6 | 100.0% |
| **GO:0016887** | 8 | 6 | 75.0% |
| **GO:0000287** | 23 | 21 | 91.3043478261% |
| **GO:0004711** | 1 | 1 | 100.0% |
| **GO:0005004** | 1 | 0 | 0.0% |
| **GO:0008046** | 1 | 0 | 0.0% |
| **GO:0004706** | 1 | 0 | 0.0% |
| **GO:0060175** | 1 | 0 | 0.0% |
| **GO:0004709** | 7 | 6 | 85.7142857143% |
| **GO:0003676** | 14 | 14 | 100.0% |
| **GO:0008234** | 4 | 4 | 100.0% |
| **GO:0005099** | 1 | 1 | 100.0% |
| **GO:0003723** | 61 | 43 | 70.4918032787% |
| **GO:0016303** | 1 | 1 | 100.0% |
| **GO:0003724** | 2 | 1 | 50.0% |
| **GO:0008186** | 1 | 0 | 0.0% |
| **GO:0004712** | 7 | 2 | 28.5714285714% |
| **GO:0046974** | 2 | 0 | 0.0% |
| **GO:0046976** | 3 | 1 | 33.3333333333% |
| **GO:0050321** | 7 | 2 | 28.5714285714% |
| **GO:0044212** | 8 | 8 | 100.0% |
| **GO:0005516** | 5 | 3 | 60.0% |
| **GO:0032403** | 5 | 4 | 80.0% |
| **GO:0051721** | 3 | 3 | 100.0% |
| **GO:0005089** | 1 | 1 | 100.0% |
| **GO:0019787** | 1 | 1 | 100.0% |
| **GO:0032947** | 2 | 0 | 0.0% |
| **GO:0035173** | 2 | 1 | 50.0% |
| **GO:0071884** | 1 | 1 | 100.0% |

| | | | |
|---|---|---|---|
| **GO:0005083** | 4 | 4 | 100.0% |
| **GO:0030676** | 1 | 1 | 100.0% |
| **GO:0004704** | 2 | 1 | 50.0% |
| **GO:0042800** | 1 | 1 | 100.0% |
| **GO:0030374** | 1 | 1 | 100.0% |
| **GO:0004525** | 1 | 1 | 100.0% |
| **GO:0035198** | 1 | 1 | 100.0% |
| **GO:0003774** | 4 | 3 | 75.0% |
| **GO:0000982** | 2 | 2 | 100.0% |
| **GO:0000166** | 62 | 62 | 100.0% |
| **GO:0004722** | 4 | 3 | 75.0% |
| **GO:0004550** | 3 | 3 | 100.0% |
| **GO:0016702** | 2 | 2 | 100.0% |
| **GO:0004708** | 7 | 5 | 71.4285714286% |
| **GO:0004298** | 15 | 15 | 100.0% |
| **GO:0008384** | 2 | 2 | 100.0% |
| **GO:0008565** | 4 | 4 | 100.0% |
| **GO:0008134** | 6 | 0 | 0.0% |
| **GO:0003690** | 11 | 4 | 36.3636363636% |
| **GO:0008301** | 5 | 0 | 0.0% |
| **GO:0008094** | 4 | 4 | 100.0% |
| **GO:0004449** | 3 | 3 | 100.0% |
| **GO:0003777** | 5 | 5 | 100.0% |
| **GO:0042623** | 3 | 2 | 66.6666666667% |
| **GO:0004872** | 3 | 2 | 66.6666666667% |
| **GO:0019789** | 1 | 1 | 100.0% |
| **GO:0003678** | 5 | 3 | 60.0% |
| **GO:0000989** | 2 | 1 | 50.0% |
| **GO:0034450** | 1 | 0 | 0.0% |
| **GO:0003727** | 3 | 3 | 100.0% |
| **GO:0004459** | 2 | 2 | 100.0% |
| **GO:0004784** | 1 | 1 | 100.0% |
| **GO:0005507** | 1 | 1 | 100.0% |
| **GO:0030346** | 1 | 1 | 100.0% |
| **GO:0004422** | 1 | 1 | 100.0% |
| **GO:0052657** | 1 | 1 | 100.0% |
| **GO:0005006** | 1 | 0 | 0.0% |
| **GO:0070888** | 5 | 5 | 100.0% |
| **GO:0003924** | 7 | 7 | 100.0% |
| **GO:0005525** | 14 | 14 | 100.0% |
| **GO:0019002** | 1 | 1 | 100.0% |
| **GO:0019003** | 2 | 2 | 100.0% |
| **GO:0005161** | 2 | 2 | 100.0% |
| **GO:0005518** | 3 | 1 | 33.3333333333% |
| **GO:0016176** | 1 | 1 | 100.0% |
| **GO:0043499** | 5 | 5 | 100.0% |
| **GO:0032395** | 2 | 2 | 100.0% |
| **GO:0042802** | 4 | 2 | 50.0% |
| **GO:0005212** | 2 | 1 | 50.0% |
| **GO:0051015** | 2 | 0 | 0.0% |
| **GO:0005506** | 2 | 2 | 100.0% |
| **GO:0008199** | 1 | 1 | 100.0% |
| **GO:0016491** | 2 | 1 | 50.0% |
| **GO:0005496** | 2 | 2 | 100.0% |
| **GO:0042562** | 1 | 0 | 0.0% |
| **GO:0004332** | 2 | 2 | 100.0% |
| **GO:0004888** | 3 | 3 | 100.0% |

| | | | |
|---|---|---|---|
| **GO:0048156** | 1 | 0 | 0.0% |
| **GO:0048306** | 1 | 1 | 100.0% |
| **GO:0070061** | 2 | 2 | 100.0% |
| **GO:0004029** | 1 | 0 | 0.0% |
| **GO:0004030** | 1 | 0 | 0.0% |
| **GO:0000049** | 1 | 0 | 0.0% |
| **GO:0004693** | 10 | 6 | 60.0% |
| **GO:0016209** | 1 | 1 | 100.0% |
| **GO:0015075** | 1 | 1 | 100.0% |
| **GO:0030246** | 2 | 2 | 100.0% |
| **GO:0030911** | 1 | 1 | 100.0% |
| **GO:0003707** | 7 | 7 | 100.0% |
| **GO:0003872** | 2 | 2 | 100.0% |
| **GO:0016208** | 2 | 1 | 50.0% |
| **GO:0008307** | 1 | 1 | 100.0% |
| **GO:0017069** | 1 | 1 | 100.0% |
| **GO:0003684** | 7 | 5 | 71.4285714286% |
| **GO:0009055** | 2 | 2 | 100.0% |
| **GO:0015035** | 2 | 2 | 100.0% |
| **GO:0015037** | 1 | 0 | 0.0% |
| **GO:0004903** | 1 | 1 | 100.0% |
| **GO:0017046** | 1 | 1 | 100.0% |
| **GO:0070064** | 1 | 0 | 0.0% |
| **GO:0003918** | 2 | 2 | 100.0% |
| **GO:0043566** | 1 | 0 | 0.0% |
| **GO:0008434** | 1 | 1 | 100.0% |
| **GO:0030674** | 1 | 0 | 0.0% |
| **GO:0050840** | 1 | 0 | 0.0% |
| **GO:0030020** | 1 | 0 | 0.0% |
| **GO:0046982** | 2 | 2 | 100.0% |
| **GO:0070644** | 1 | 0 | 0.0% |
| **GO:0000980** | 2 | 2 | 100.0% |
| **GO:0070095** | 1 | 0 | 0.0% |
| **GO:0017111** | 2 | 2 | 100.0% |
| **GO:0042162** | 1 | 1 | 100.0% |
| **GO:0042277** | 1 | 0 | 0.0% |
| **GO:0043138** | 2 | 0 | 0.0% |
| **GO:0000403** | 3 | 1 | 33.3333333333% |
| **GO:0000406** | 2 | 1 | 50.0% |
| **GO:0019237** | 2 | 0 | 0.0% |
| **GO:0005215** | 2 | 2 | 100.0% |
| **GO:0004145** | 2 | 0 | 0.0% |
| **GO:0019809** | 1 | 1 | 100.0% |
| **GO:0019838** | 1 | 0 | 0.0% |
| **GO:0004719** | 1 | 1 | 100.0% |
| **GO:0008168** | 1 | 1 | 100.0% |
| **GO:0008641** | 1 | 1 | 100.0% |
| **GO:0030332** | 1 | 0 | 0.0% |
| **GO:0003730** | 3 | 1 | 33.3333333333% |
| **GO:0017091** | 1 | 0 | 0.0% |
| **GO:0036002** | 1 | 0 | 0.0% |
| **GO:0015026** | 1 | 1 | 100.0% |
| **GO:0016362** | 1 | 1 | 100.0% |
| **GO:0004175** | 2 | 0 | 0.0% |
| **GO:0042626** | 1 | 1 | 100.0% |
| **GO:0004896** | 2 | 2 | 100.0% |
| **GO:0050897** | 1 | 1 | 100.0% |

| | | | |
|---|---|---|---|
| **GO:0003746** | 1 | 1 | 100.0% |
| **GO:0004392** | 1 | 1 | 100.0% |
| **GO:0015631** | 1 | 0 | 0.0% |
| **GO:0046933** | 1 | 1 | 100.0% |
| **GO:0046961** | 1 | 1 | 100.0% |
| **GO:0003735** | 5 | 5 | 100.0% |
| **GO:0032137** | 3 | 3 | 100.0% |
| **GO:0038046** | 1 | 1 | 100.0% |
| **GO:0008080** | 1 | 1 | 100.0% |
| **GO:0002161** | 1 | 1 | 100.0% |
| **GO:0004822** | 1 | 1 | 100.0% |
| **GO:0000983** | 1 | 0 | 0.0% |
| **GO:0000404** | 1 | 1 | 100.0% |
| **GO:0004886** | 1 | 0 | 0.0% |
| **GO:0043035** | 1 | 1 | 100.0% |
| **GO:0003688** | 1 | 1 | 100.0% |
| **GO:0002039** | 1 | 0 | 0.0% |
| **GO:0015068** | 1 | 1 | 100.0% |
| **GO:0016813** | 1 | 1 | 100.0% |
| **GO:0016493** | 1 | 1 | 100.0% |
| **GO:0000340** | 1 | 1 | 100.0% |
| **GO:0003899** | 1 | 1 | 100.0% |
| **GO:0001104** | 1 | 1 | 100.0% |
| **GO:0001190** | 1 | 1 | 100.0% |
| **GO:0003705** | 3 | 3 | 100.0% |
| **GO:0010736** | 1 | 1 | 100.0% |
| **GO:0004814** | 1 | 1 | 100.0% |
| **GO:0004691** | 2 | 0 | 0.0% |
| **GO:0008603** | 1 | 1 | 100.0% |
| **GO:0043531** | 2 | 1 | 50.0% |
| **GO:0004679** | 2 | 2 | 100.0% |
| **GO:0035174** | 2 | 2 | 100.0% |
| **GO:0047322** | 2 | 1 | 50.0% |
| **GO:0050405** | 2 | 1 | 50.0% |
| **GO:0016616** | 2 | 2 | 100.0% |
| **GO:0000978** | 1 | 1 | 100.0% |
| **GO:0004438** | 1 | 1 | 100.0% |
| **GO:0004725** | 1 | 1 | 100.0% |
| **GO:0008138** | 1 | 1 | 100.0% |
| **GO:0016314** | 1 | 1 | 100.0% |
| **GO:0051717** | 1 | 1 | 100.0% |
| **GO:0051800** | 1 | 1 | 100.0% |
| **GO:0004749** | 1 | 1 | 100.0% |
| **GO:0016301** | 1 | 1 | 100.0% |
| **GO:0017137** | 1 | 1 | 100.0% |
| **GO:0016494** | 1 | 1 | 100.0% |
| **GO:0018169** | 1 | 1 | 100.0% |
| **GO:0019788** | 1 | 1 | 100.0% |
| **GO:0019843** | 1 | 1 | 100.0% |
| **GO:0005344** | 1 | 1 | 100.0% |
| **GO:0019825** | 1 | 1 | 100.0% |
| **GO:0020037** | 1 | 1 | 100.0% |
| **GO:0005092** | 1 | 1 | 100.0% |
| **GO:0070363** | 1 | 1 | 100.0% |
| **GO:0030549** | 1 | 1 | 100.0% |
| **GO:0000975** | 1 | 1 | 100.0% |
| **GO:0004728** | 1 | 1 | 100.0% |

| | | | |
|---|---|---|---|
| **GO:0042605** | 1 | 0 | 0.0% |
| **GO:0046978** | 1 | 0 | 0.0% |
| **GO:0046979** | 1 | 0 | 0.0% |
| **GO:0046980** | 1 | 1 | 100.0% |
| **GO:0043142** | 1 | 0 | 0.0% |
| **GO:0005102** | 1 | 1 | 100.0% |
| **GO:0008379** | 2 | 2 | 100.0% |
| **GO:0004879** | 1 | 1 | 100.0% |
| **GO:0008144** | 1 | 0 | 0.0% |
| **GO:0019904** | 2 | 0 | 0.0% |
| **GO:0070053** | 1 | 1 | 100.0% |
| **GO:0008599** | 1 | 1 | 100.0% |
| **GO:0043539** | 2 | 2 | 100.0% |
| **GO:0046983** | 1 | 1 | 100.0% |
| **GO:0016881** | 2 | 2 | 100.0% |
| **GO:0000987** | 1 | 1 | 100.0% |
| **GO:0030616** | 1 | 1 | 100.0% |
| **GO:0019901** | 1 | 0 | 0.0% |
| **GO:0030971** | 1 | 0 | 0.0% |
| **GO:0042169** | 1 | 1 | 100.0% |
| **GO:0043130** | 1 | 1 | 100.0% |
| **GO:0004683** | 4 | 3 | 75.0% |
| **GO:0051010** | 1 | 1 | 100.0% |
| **GO:0017049** | 2 | 2 | 100.0% |
| **GO:0043008** | 1 | 0 | 0.0% |
| **GO:0030371** | 1 | 1 | 100.0% |
| **GO:0070181** | 1 | 0 | 0.0% |
| **GO:0008060** | 1 | 1 | 100.0% |
| **GO:0004835** | 1 | 1 | 100.0% |
| **GO:0008408** | 1 | 1 | 100.0% |
| **GO:0042054** | 2 | 1 | 50.0% |
| **GO:0005545** | 2 | 2 | 100.0% |
| **GO:0030742** | 1 | 0 | 0.0% |
| **GO:0018024** | 1 | 1 | 100.0% |
| **GO:0005097** | 1 | 1 | 100.0% |
| **GO:0005244** | 1 | 1 | 100.0% |
| **GO:0005267** | 1 | 1 | 100.0% |
| **GO:0004721** | 1 | 0 | 0.0% |
| **GO:0030295** | 2 | 2 | 100.0% |
| **GO:0005547** | 1 | 1 | 100.0% |
| **GO:0043021** | 1 | 1 | 100.0% |
| **GO:0010484** | 1 | 1 | 100.0% |
| **GO:0043997** | 1 | 1 | 100.0% |
| **GO:0004252** | 1 | 1 | 100.0% |
| **GO:0004475** | 1 | 1 | 100.0% |
| **GO:0034584** | 1 | 1 | 100.0% |
| **GO:0004864** | 1 | 1 | 100.0% |
| **GO:0000104** | 1 | 1 | 100.0% |
