# Supplementary material for: A combined approach for genome wide protein function annotation/prediction
Source: Proteome Sci. 2013 Nov 7;11(Suppl 1):S1. doi: 10.1186/1477-5956-11-S1-S1 (PMC3909112; doi:10.1186/1477-5956-11-S1-S1)
Supplement: Additional file 2 — Term wise prediction-Cerevisiae.pdf Term wise prediction results for Saccharomyces Cerevisiae data set. [file 1477-5956-11-S1-S1-S2.pdf]

Gene Ontology Function	Total Appearance Count	Total Prediction Count	Term Coverage
**GO:0008270**	65	65	100.0%
**GO:0000166**	51	51	100.0%
**GO:0004365**	3	3	100.0%
**GO:0050661**	5	5	100.0%
**GO:0051287**	12	12	100.0%
**GO:0004693**	5	1	20.0%
**GO:0005524**	258	258	100.0%
**GO:0042393**	2	0	0.0%
**GO:0000287**	25	25	100.0%
**GO:0004743**	2	2	100.0%
**GO:0030955**	2	2	100.0%
**GO:0004427**	2	2	100.0%
**GO:0008553**	1	1	100.0%
**GO:0046933**	2	2	100.0%
**GO:0046961**	4	4	100.0%
**GO:0004634**	2	2	100.0%
**GO:0004825**	1	1	100.0%
**GO:0003924**	18	18	100.0%
**GO:0005525**	21	21	100.0%
**GO:0003677**	39	33	84.6153846154%
**GO:0005200**	1	1	100.0%
**GO:0003746**	5	5	100.0%
**GO:0004088**	2	1	50.0%
**GO:0046872**	30	30	100.0%
**GO:0005471**	2	2	100.0%
**GO:0004396**	1	1	100.0%
**GO:0000981**	10	7	70.0%
**GO:0004585**	1	0	0.0%
**GO:0016597**	2	1	50.0%
**GO:0003747**	1	1	100.0%
**GO:0004691**	3	3	100.0%
**GO:0003743**	5	5	100.0%
**GO:0004737**	3	3	100.0%
**GO:0030976**	5	5	100.0%
**GO:0003852**	2	2	100.0%
**GO:0004674**	64	64	100.0%
**GO:0004004**	14	11	78.5714285714%
**GO:0033592**	1	0	0.0%
**GO:0004679**	1	0	0.0%
**GO:0004708**	4	0	0.0%
**GO:0004637**	1	1	100.0%
**GO:0004641**	1	1	100.0%
**GO:0004329**	2	2	100.0%
**GO:0004488**	2	2	100.0%
**GO:0004354**	2	0	0.0%
**GO:0004828**	1	1	100.0%
**GO:0003984**	1	1	100.0%
**GO:0050660**	3	3	100.0%
**GO:0002161**	3	3	100.0%
**GO:0004832**	1	1	100.0%
**GO:0004713**	6	0	0.0%
**GO:0003899**	8	8	100.0%
**GO:0032549**	3	3	100.0%
**GO:0000146**	4	2	50.0%

**GO:0016887**	19	16	84.2105263158%
**GO:0051082**	19	18	94.7368421053%
**GO:0004822**	1	1	100.0%
**GO:0004748**	4	4	100.0%
**GO:0046914**	2	2	100.0%
**GO:0005515**	35	34	97.1428571429%
**GO:0008026**	14	14	100.0%
**GO:0005506**	2	2	100.0%
**GO:0009055**	5	4	80.0%
**GO:0020037**	2	2	100.0%
**GO:0003700**	4	3	75.0%
**GO:0043565**	11	5	45.4545454545%
**GO:0008301**	3	1	33.3333333333%
**GO:0031491**	3	2	66.6666666667%
**GO:0004222**	3	3	100.0%
**GO:0005388**	1	1	100.0%
**GO:0005509**	9	8	88.8888888889%
**GO:0030983**	7	7	100.0%
**GO:0004712**	4	1	25.0%
**GO:0004707**	4	4	100.0%
**GO:0004721**	3	2	66.6666666667%
**GO:0003755**	5	5	100.0%
**GO:0005487**	6	5	83.3333333333%
**GO:0017056**	6	6	100.0%
**GO:0003676**	17	17	100.0%
**GO:0004824**	1	1	100.0%
**GO:0000049**	3	2	66.6666666667%
**GO:0004694**	1	0	0.0%
**GO:0004826**	1	1	100.0%
**GO:0051015**	1	1	100.0%
**GO:0008186**	3	0	0.0%
**GO:0019843**	5	2	40.0%
**GO:0003872**	2	2	100.0%
**GO:0004520**	1	0	0.0%
**GO:0030060**	2	2	100.0%
**GO:0004340**	2	0	0.0%
**GO:0005198**	7	6	85.7142857143%
**GO:0004672**	23	8	34.7826086957%
**GO:0005099**	1	0	0.0%
**GO:0003723**	58	47	81.0344827586%
**GO:0015171**	4	4	100.0%
**GO:0015220**	1	0	0.0%
**GO:0034228**	1	0	0.0%
**GO:0004722**	7	2	28.5714285714%
**GO:0004298**	14	14	100.0%
**GO:0003951**	2	2	100.0%
**GO:0042736**	2	0	0.0%
**GO:0004386**	13	13	100.0%
**GO:0008094**	8	7	87.5%
**GO:0003688**	7	7	100.0%
**GO:0004802**	2	2	100.0%
**GO:0008353**	2	0	0.0%
**GO:0004373**	2	2	100.0%
**GO:0005488**	4	4	100.0%
**GO:0004709**	2	0	0.0%
**GO:0005315**	1	1	100.0%
**GO:0005507**	2	2	100.0%

**GO:0042623**	1	1	100.0%
**GO:0003682**	10	6	60.0%
**GO:0004697**	1	0	0.0%
**GO:0003724**	5	2	40.0%
**GO:0019901**	3	3	100.0%
**GO:0004175**	4	3	75.0%
**GO:0004725**	3	2	66.6666666667%
**GO:0003729**	6	4	66.6666666667%
**GO:0051087**	1	0	0.0%
**GO:0000406**	2	1	50.0%
**GO:0003684**	4	4	100.0%
**GO:0032135**	1	0	0.0%
**GO:0010181**	5	5	100.0%
**GO:0016491**	4	4	100.0%
**GO:0015193**	2	2	100.0%
**GO:0003887**	5	5	100.0%
**GO:0003964**	5	5	100.0%
**GO:0004190**	6	6	100.0%
**GO:0004523**	5	5	100.0%
**GO:0004540**	5	5	100.0%
**GO:0008233**	5	5	100.0%
**GO:0000150**	1	0	0.0%
**GO:0003697**	3	0	0.0%
**GO:0030515**	5	5	100.0%
**GO:0043130**	5	3	60.0%
**GO:0000400**	2	1	50.0%
**GO:0004724**	1	1	100.0%
**GO:0048037**	1	1	100.0%
**GO:0004823**	1	1	100.0%
**GO:0004683**	1	0	0.0%
**GO:0004449**	2	2	100.0%
**GO:0003883**	2	2	100.0%
**GO:0050291**	2	2	100.0%
**GO:0004652**	1	1	100.0%
**GO:0004791**	2	2	100.0%
**GO:0009378**	3	3	100.0%
**GO:0008565**	7	7	100.0%
**GO:0004871**	3	3	100.0%
**GO:0005484**	11	11	100.0%
**GO:0004169**	4	4	100.0%
**GO:0043531**	1	0	0.0%
**GO:0004075**	3	3	100.0%
**GO:0004736**	1	1	100.0%
**GO:0008271**	1	0	0.0%
**GO:0034658**	1	0	0.0%
**GO:0004555**	2	2	100.0%
**GO:0005353**	3	3	100.0%
**GO:0005354**	1	0	0.0%
**GO:0005355**	3	3	100.0%
**GO:0015146**	2	1	50.0%
**GO:0015578**	3	3	100.0%
**GO:0004847**	1	0	0.0%
**GO:0016884**	1	1	100.0%
**GO:0008559**	4	3	75.0%
**GO:0001104**	1	1	100.0%
**GO:0004221**	6	6	100.0%
**GO:0008266**	1	0	0.0%

**GO:0015616**	2	0	0.0%
**GO:0070577**	3	3	100.0%
**GO:0008144**	2	2	100.0%
**GO:0030619**	2	0	0.0%
**GO:0015035**	2	2	100.0%
**GO:0015036**	2	2	100.0%
**GO:0004012**	3	3	100.0%
**GO:0015662**	4	4	100.0%
**GO:0003779**	1	1	100.0%
**GO:0030674**	2	0	0.0%
**GO:0042802**	1	0	0.0%
**GO:0004749**	5	5	100.0%
**GO:0016301**	5	5	100.0%
**GO:0003735**	2	2	100.0%
**GO:0032266**	4	3	75.0%
**GO:0017111**	4	4	100.0%
**GO:0004614**	2	2	100.0%
**GO:0042626**	2	2	100.0%
**GO:0008379**	3	3	100.0%
**GO:0004003**	2	0	0.0%
**GO:0043140**	1	1	100.0%
**GO:0004402**	2	1	50.0%
**GO:0003999**	2	2	100.0%
**GO:0003937**	2	2	100.0%
**GO:0004643**	2	2	100.0%
**GO:0004820**	1	1	100.0%
**GO:0046983**	1	0	0.0%
**GO:0004032**	2	2	100.0%
**GO:0032947**	1	0	0.0%
**GO:0043142**	1	1	100.0%
**GO:0000182**	1	0	0.0%
**GO:0043169**	2	2	100.0%
**GO:0003824**	5	5	100.0%
**GO:0030234**	2	0	0.0%
**GO:0003938**	4	4	100.0%
**GO:0004816**	1	1	100.0%
**GO:0004827**	1	1	100.0%
**GO:0004616**	2	2	100.0%
**GO:0000156**	2	2	100.0%
**GO:0004084**	2	2	100.0%
**GO:0052654**	2	2	100.0%
**GO:0052655**	2	2	100.0%
**GO:0052656**	2	2	100.0%
**GO:0005528**	2	2	100.0%
**GO:0000989**	1	0	0.0%
**GO:0009922**	1	1	100.0%
**GO:0008092**	1	1	100.0%
**GO:0008237**	2	2	100.0%
**GO:0004617**	2	1	50.0%
**GO:0008519**	2	2	100.0%
**GO:0047046**	1	0	0.0%
**GO:0008143**	2	2	100.0%
**GO:0043138**	1	0	0.0%
**GO:0030620**	1	0	0.0%
**GO:0004864**	1	1	100.0%
**GO:0004367**	2	2	100.0%
**GO:0004450**	1	1	100.0%

**GO:0004029**	2	0	0.0%
**GO:0004030**	2	0	0.0%
**GO:0004818**	1	1	100.0%
**GO:0043022**	1	1	100.0%
**GO:0005543**	3	3	100.0%
**GO:0004410**	2	1	50.0%
**GO:0004066**	2	2	100.0%
**GO:0016787**	1	0	0.0%
**GO:0017070**	2	0	0.0%
**GO:0005089**	2	2	100.0%
**GO:0004842**	1	1	100.0%
**GO:0000297**	3	3	100.0%
**GO:0015297**	3	3	100.0%
**GO:0019904**	1	0	0.0%
**GO:0016874**	1	1	100.0%
**GO:0004497**	1	1	100.0%
**GO:0008138**	2	2	100.0%
**GO:0003989**	1	1	100.0%
**GO:0008142**	2	2	100.0%
**GO:0070300**	1	0	0.0%
**GO:0042162**	1	1	100.0%
**GO:0016740**	1	1	100.0%
**GO:0035174**	1	0	0.0%
**GO:0008276**	1	0	0.0%
**GO:0008757**	1	0	0.0%
**GO:0008234**	3	3	100.0%
**GO:0004814**	1	1	100.0%
**GO:0033680**	1	0	0.0%
**GO:0008972**	3	3	100.0%
**GO:0015606**	1	0	0.0%
**GO:0008902**	2	0	0.0%
**GO:0008097**	1	0	0.0%
**GO:0042134**	1	1	100.0%
**GO:0003910**	1	1	100.0%
**GO:0016616**	1	1	100.0%
**GO:0000339**	1	0	0.0%
**GO:0036002**	1	0	0.0%
**GO:0042803**	1	0	0.0%
